# Palliative Care and Oncology in Colombia: The Potential of Integrated Care Delivery

**DOI:** 10.3390/healthcare9070789

**Published:** 2021-06-23

**Authors:** Joanne Reid, Esther de Vries, Sam H. Ahmedzai, Mauricio Arias-Rojas, Jose Andrés Calvache, Susana Carolina Gómez-Sarmiento, Monica Lucia Gomez-Serrano, Sandra Liliana Parra-Cubides, Gillian Prue, Socorro Moreno Luna

**Affiliations:** 1 Medical Biology Centre, School of Nursing and Midwifery, Queen’s University Belfast, Belfast BT9 7BL, UK; g.prue@qub.ac.uk; 2 Department of Clinical Epidemiology and Biostatistics, Pontificia Universidad Javeriana, Bogotá 110231, Colombia; estherdevries@javeriana.edu.co (E.d.V.); Isabel.moreno@javeriana.edu.co (S.M.L.); 3 Medical School, The University of Sheffield, Beech Hill Road, Sheffield S10 2RX, UK; s.ahmedzai@sheffield.ac.uk; 4 Faculty of Nursing, Universidad de Antioquia, Medellín 050010, Colombia; emauricio.arias@udea.edu.co; 5 Department of Anesthesiology, Universidad del Cauca, Popayán 760032, Colombia; jacalvache@unicauca.edu.co; 6 Pain and Palliative Care Physician, Liga Colombiana Contra el Cáncer, Bogotá 110231, Colombia; cuidadospaliativos@ligacancercolombia.org; 7 Holistic Volunteering Support (VIDA), the Colombian Cancer League (Liga Colombiana Contra el Cáncer), Bogotá 110231, Colombia; matana44@yahoo.es; 8 Palliative Care and Pain Medicine Service, Clínica El Rosario, Medellín 050010, Colombia; dolor@clinicaelrosario.com

Palliative care is on the global health agenda, as only approximately 14% of people who require palliative care receive it [1]. The World Health Organisation (WHO) defines palliative care as a care approach that “improves the quality of life of patients and that of their families who are facing challenges associated with life-threatening illness, whether physical, psychological, social or spiritual. The quality of life of caregivers improves as well”. To achieve universal health coverage, a core component of the United Nation’s sustainable development goal 3 (“Ensure healthy lives and promote well-being for all at all ages”), palliative care is a prerequisite [2,3]. Several randomised trials in cancer populations have demonstrated that early access to palliative care has significant benefits: for patients in terms of symptom burden, quality of life, end-of-life care [4,5,6,7,8]; for the healthcare system in terms of reduced financial burden for both patients and the health system [9]; and for informal family caregivers in terms of stress burden and depression [10]. A multinational consensus across Europe identified that palliative care should be delivered at both the “basic” and “specialised” levels, and called for recognition of palliative care in healthcare systems, including within oncology and primary care [11]. However, a recent survey of patients with advanced cancer identified unmet palliative care needs in nearly two thirds of patients [12], and some authors argue that palliative care is still in its infancy in most of the developing world [13].

Colombia is a middle-income country in South America with a population of around 50 million inhabitants. The population aged 65 and over is projected to rise between 2019–2030 from 4,413,000 (8.8%) to 6,962,000 (13%) [14], with life expectancy growing rapidly (from 72.9 in 2000 to 77.1 years in 2018) [15]. However, cancer survival remains relatively poor [16], implying that a rapidly increasing number of cancer patients, as well as patients suffering from other chronic conditions, such as cardiovascular disease and chronic respiratory diseases [17], are in need of palliative care. This has led to a call for geriatric medicine to also embrace the future need for palliative care in the growing frail older population [18]. Moreover, it is estimated that 78% of the 40 million people who require palliative care globally reside in low- and middle-income countries (LMICs) [1].

In 2015, the Lancet Oncology Commission recommended that cancer control in Latin America required the full range of services, including palliative care [19], and the 2018 Lancet Oncology Commission on palliative care focusing on LMICs highlighted the inequity that exists internationally in relation to people who are poor, living and dying with little or no access to palliative care service provision [20]. The Colombian Ministry of Health and Social Protection has oversight of regulating the mandatory health insurers (EPS) and the care providers (IPS) to ensure that their service network provides comprehensive care in palliative care “according to the level of complexity”. As of 2014, Colombia has organised legislation regarding the availability of palliative care with the aim of guaranteeing access to palliative care to all patients who may benefit from such care, including those with cancer and non-cancer diseases [21]. Nevertheless, the offer of palliative care services in the country is still scarce and mainly limited to hospital-based settings and centralised in large cities [22,23,24,25], despite the fact that throughout the country, there was an increase in palliative care services from 23 in 2013 to 79 in 2020. This increase in service provision is still insufficient for the needs of the population [26], whereas the global atlas of palliative care outlined Colombia [27] as having generalised palliative care provision [28]. The rising demand for palliative care and both personal and financial implications of this, particularly in Colombia [29], highlight the need for reconfiguration of cancer services to integrate and provide palliative care alongside existing healthcare provision. Home-based palliative care services are very rare, but they have been identified by healthcare professionals as very important [30].

Integrated care is viewed as an important framework and organising principle, enhancing the quality of care delivery with the aim of achieving improved patient care through better coordination of services. The WHO defines integrated health services as “health services that are managed and delivered so that people receive a continuum of health promotion, disease prevention, diagnosis, treatment, disease-management, rehabilitation and palliative care services, coordinated across the different levels and sites of care within and beyond the health sector, and according to their needs throughout the life course.” [31](p. 2). Research that currently exists in relation to integrating palliative care and oncology is heterogeneous. The majority of studies have been conducted in high-income countries, and within these, there is variation across nations, healthcare settings and associated systems, which greatly limit any generalisability from such work [32]. In its simplest terms, integrated care is “the right care, at the right time, in the right place, by the right person” [33](p. 8). As early as the new millennium, there was a call for the emerging specialty of palliative medicine to become incorporated into cancer care [34]. However, the complexity of integrating palliative care and oncology is well documented [32], and there is as yet no integrative oncology palliative care model [35] to optimise the delivery of high-quality comprehensive care for patients. This is despite a growing consensus for integration of oncology and palliative care and empirical evidence highlighting the benefits of such [4,5,6,7,8,9,10,36,37]. Indeed, a recent Lancet commission in relation to oncology and palliative care did not identify any healthcare system where the content and the constructs of integration are implemented [32]. This highlights that there is currently no singular agreed model of integration that can be implemented. To produce evidence in this area, work is required to synthesise a detailed understanding of which integrated palliative care and cancer interventions may work best, for whom and in what circumstances. Additionally, the importance of being cognisant of factors such as local organisational, cultural and health policy aspects in relation to any model of integration has been outlined in the literature [38].

Despite marked developments in palliative care in Colombia over this last decade, there remains fragmentation between oncology and palliative care, hampering the provision of optimal care. The challenges that exist in relation to patient/carer needs as well as access to and provision of palliative care for people with cancer have not been robustly studied in LMICs, or in the local context of Colombia. Additionally, a recent systematic review focusing on the implementation of cancer treatment and palliative care strategies in LMICs confirmed the importance of having stakeholder engagement to co-design and implement quality care [39]. This is also reflected in the NICE Guidance on Cancer Services Improving Supportive and Palliative Care for Adults with Cancer [40]. Such stakeholders would include service users and carers, statutory and voluntary service providers and policy makers to develop an inclusive model of care that recognises and responds to the needs of this client group. Central to involving key stakeholders is the evaluation of any such a model to ensure any benefits of integration in relation to systems, patient/carer experience and outcomes are appropriately captured [41]. Thus, future research is warranted to develop an evidence-based integrated model of palliative oncology care, which should place value on the role of family medicine and geriatrics alongside oncology. Previous work has highlighted that the majority of palliative care research to date has been conducted in high-income countries [42], and there is a requirement for empirical data from LMICs to develop the evidence base to guide both policy and practice, which recognises and responds to the needs to this client cohort.
